# A Phase I Evaluation of the Pharmacokinetics and Tolerability of the HIV-1 Maturation Inhibitor GSK3640254 and Tenofovir Alafenamide/Emtricitabine in Healthy Participants

**DOI:** 10.1128/AAC.02173-20

**Published:** 2021-05-18

**Authors:** Teodora Pene Dumitrescu, Samit R. Joshi, Jianfeng Xu, Joyce Zhan, Mark Johnson, Laurie Butcher, Eric Zimmerman, Lindsey Webster, Antonia M. Davidson, Max Lataillade, Sherene Min

**Affiliations:** a GlaxoSmithKline, Collegeville, Pennsylvania, USA; b ViiV Healthcare, Branford, Connecticut, USA; c ViiV Healthcare, Research Triangle Park, North Carolina, USA; d PPD, Richmond, Virginia, USA; e PPD, Austin, Texas, USA

**Keywords:** HIV-1 infection, clinical study, antiretroviral, pharmacokinetic

## Abstract

GSK3640254 is a next-generation maturation inhibitor that would likely be combined with standard antiretroviral agents to form a regimen of ≥2 fully active classes. This phase I, open-label, 2-period, 1-way study assessed potential pharmacokinetic (PK) interactions between GSK3640254 and tenofovir alafenamide/emtricitabine (TAF/FTC; including the metabolite tenofovir [TFV]) in healthy volunteers. Eligible participants received TAF/FTC 25/200 mg once daily (QD) on days 1 through 21 with a moderate-fat meal; GSK3640254 200 mg QD was added on days 15 through 21. Geometric least-squares mean ratios (GMRs) and 90% confidence intervals (CIs) were derived using linear mixed-effect models. Adverse events (AEs) and laboratory, electrocardiogram, and vital sign parameters were monitored. Sixteen participants, all male, received treatment; one withdrew because of treatment-related grade 1 urticaria. After TAF/FTC + GSK3640254 coadministration, TAF steady-state area under the plasma concentration-time curve from time zero to the end of the dosing interval and maximum observed concentration were 11% and 13% lower than when TAF/FTC was administered alone, with GMRs (90% CI) of 0.886 (0.75 to 1.04) and 0.874 (0.68 to 1.12), respectively. Steady-state PK of TFV and FTC was similar when TAF/FTC was administered alone or with GSK3640254. No clinically significant trends in tolerability or safety were observed. GSK3640254 200 mg QD did not meaningfully affect the steady-state PK of TAF, TFV, or FTC in healthy participants under fed conditions and was not associated with major tolerability or safety findings. These data support the further investigation of GSK3640254 for coadministration with TAF/FTC for the treatment of HIV. (This study has been registered at ClinicalTrials.gov under identifier NCT03836729.)

## TEXT

Current antiretroviral therapy regimens for HIV-1 mostly target the reverse transcriptase, protease, or integrase proteins (1). However, despite recent advancements in antiretroviral therapy, drug resistance and intolerability may occur, which can lead to treatment failure (1, 2). There is a need for new antiretroviral agents to address emerging drug resistance and long-term safety and toxicity of existing agents (3).

Maturation is the last phase in the HIV-1 viral life cycle and refers to the step catalyzed by the viral protease, wherein the HIV-1 structural protein (Gag) polyprotein is cleaved into its component proteins (3). This process is essential for the newly released viral particles to become infectious and initiate the next round of infection. Therefore, viral maturation is a promising target for therapeutic intervention, as disruption of HIV-1 maturation results in production of noninfectious viral particles. Novel therapeutic agents, termed maturation inhibitors (MIs), inhibit protease-mediated cleavage of capsid (CA)-spacer protein 1 in the Gag polyprotein (3). Findings from *in vitro* analyses and phase II clinical studies in combination with other antiretroviral agents suggest that pharmacological inhibition of maturation inhibits replication of HIV-1 isolates, resulting in clinical benefit (2–6).

GSK3640254 is a novel, next-generation MI that blocks the final protease cleavage event between CA protein p24 and CA-spacer 1, resulting in noninfectious virions (7). GSK3640254 has no cross-resistance to currently approved classes, is effective against a broad range of Gag polymorphisms, and has a preclinical profile that supports clinical evaluation for treatment of HIV-1 infection. Two randomized phase I clinical trials (NCT03231943 and NCT03575962) were conducted to assess the safety, pharmacokinetics (PK), and relative bioavailability of GSK3640254 in healthy adults. GSK3640254 was slowly absorbed, with a median time to maximum concentration (*T*_max_) between 3.0 and 4.5 h and a mean half-life of ∼24 h, and was well tolerated in single and multiple ascending doses. Because antiretroviral drugs are often administered with other therapies, it is important to characterize any potential interactions or changes in exposure when medications are given in combination. GSK3640254 could potentially be coadministered with the nucleoside reverse transcriptase inhibitors tenofovir alafenamide (TAF) and emtricitabine (FTC) (8). GSK3640254 is an inhibitor of organic anion-transporting polypeptide 1B3 (OATP1B3), and *in vitro* studies have suggested the potential for interactions between GSK3640254 and substrates of OATP1B3, such as TAF (8). However, as TAF and FTC are primarily excreted by the kidneys (8), the risk of meaningful drug-drug interactions was expected to be low. Here, we report the PK and safety and tolerability from a drug interaction study of GSK3640254 and TAF/FTC in healthy participants.

## RESULTS

### Participant disposition and baseline characteristics.

A total of 40 individuals were screened, 16 participants were enrolled, and 15 participants (94%) completed the study. One participant withdrew because of a treatment-related adverse event (AE) of urticaria (described below). All 16 participants were male, with mean age of 33.9 years and mean body mass index of 26.9 kg/m^2^. The proportions of black and white participants were 44% and 38%, respectively (Table 1).

**TABLE 1:** Baseline demographics

Parameter	Value for participants (*n* = 16)
Age, mean (SD), yr	33.9 (9.7)
No. (%) of males	16 (100)
Body mass index, mean (SD), kg/m^2^	26.9 (2.0)
Ht, mean (SD), cm	176.5 (5.9)
Wt, mean (SD), kg	83.9 (7.5)
No. (%) with ethnicity
Hispanic or Latino	5 (31)
Not Hispanic or Latino	11 (69)
No. (%) with race
American Indian or Alaska Native	1 (6)
Asian/East Asian heritage	1 (6)
Black or African American	7 (44)
Native Hawaiian or other Pacific Islander	1 (6)
White	6 (38)

### PK results.

**FIG 1:**
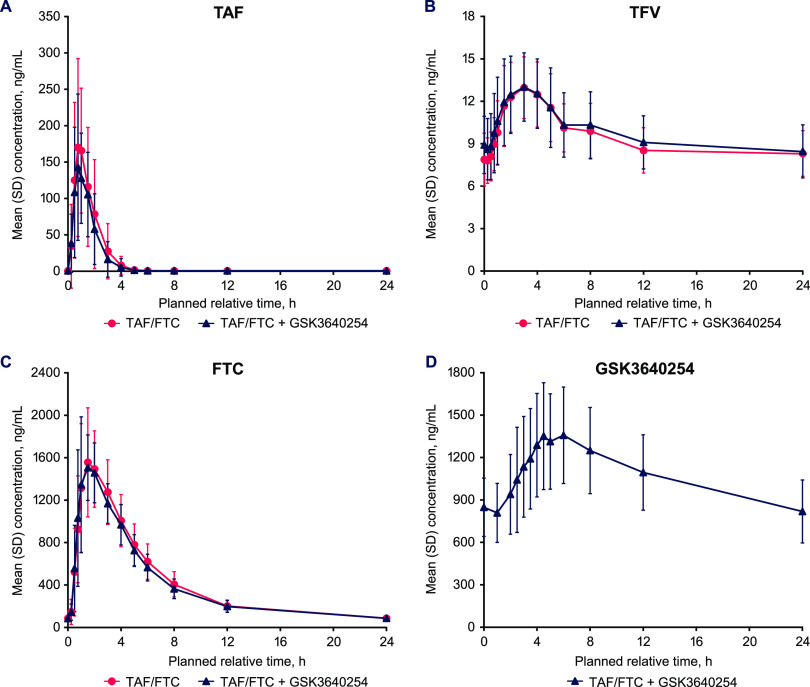
Mean linear plasma concentration-time profiles at steady state for (A) TAF, (B) TFV, (C) FTC, and (D) GSK3640254. Error bars represent standard deviations (SD). FTC, emtricitabine; TAF, tenofovir alafenamide; TFV, tenofovir.

Plasma PK parameters for TAF, tenofovir (TFV), and FTC after TAF/FTC administration and coadministration of TAF/FTC plus GSK3640254 are summarized in Table 2, and mean linear steady-state plasma concentration-time profiles are shown in Fig. 1 (semilogarithmic plots are shown in Fig. S1 and linear plots of plasma trough concentration-time profiles are shown in Fig. S2 in the supplemental material). After TAF/FTC + GSK3640254 coadministration, the TAF steady-state area under the concentration-time curve over one dosing interval (AUC_0–τ_) and maximum concentration of drug in serum (*C*_max_) were 11% and 13% lower than those for TAF/FTC administered alone. The primary analyses of interest were the geometric least-squares mean ratios (GMRs) (90% confidence intervals [CIs]) for TAF, TFV, and FTC steady-state AUC_0–τ_ and *C*_max_ between TAF/FTC + GSK3640254 coadministration and TAF/FTC administered alone, which were 0.886 (0.75 to 1.04) and 0.874 (0.68 to 1.12) for TAF AUC_0–τ_ and *C*_max_, respectively; 1.036 (1.01-1.07) and 1.018 (0.97-1.07) for TFV AUC_0–τ_ and *C*_max_, respectively; and 0.963 (0.93-1.00) and 0.941 (0.84-1.05) for FTC AUC_0–τ_ and *C*_max_, respectively (Table 3). Between-participant variability (%CVb) for exposure parameters AUC_0–τ_ and *C*_max_ was moderate (range, 36.3% to 58.2% across treatments). Plasma concentrations for TAF peaked with a median *T*_max_ of 1.0 h after dose administration for both treatments (Fig. 1A) and then rapidly declined in a monophasic manner, reaching undetectable levels within 8 h after dosing.

**TABLE 2:** Steady-state plasma PK parameters^*a*^

Drug and group	AUC_0–τ_			*C*_max_			*C*_τ_			*T*_max_, median (range), h
	Geometric mean, ng·h/ml (SD ln)	95% CI	%CVb	Geometric mean, ng/ml (SD ln)	95% CI	%CVb	Geometric mean, ng/ml (SD ln)	95% CI	%CVb	
TAF
TAF/FTC (*n* = 16)	250.4 (1.72)	187.8–333.8	58.2	203.4 (1.69)	153.9–268.8	56.1	ND	ND	ND	1.00 (0.50–2.00)
TAF/FTC + GSK3640254 (*n* = 15)	215.4 (1.42)	177.3–261.7	36.3	175.1 (1.53)	138.5–221.4	44.4	ND	ND	ND	1.00 (0.50–2.00)

TFV
TAF/FTC (*n* = 16)	221.9 (1.20)	201.4–244.4	18.3	13.1 (1.19)	12.0–14.4	17.8	7.7 (1.22)	6.9–8.6	20.4	3.00 (1.50–4.00)
TAF/FTC + GSK3640254 (*n* = 15)	229.1 (1.24)	203.6–257.7	21.5	13.3 (1.23)	11.9–14.9	20.8	8.2 (1.25)	7.3–9.3	22.9	3.00 (1.00–5.00)

FTC
TAF/FTC (*n* = 16)	9,787.5 (1.17)	9,013.9–10,627.5	15.5	1,811 (1.17)	1,665.2–1,969.6	15.9	71.8 (1.29)	62.8–82.1	25.5	1.50 (1.00–3.00)
TAF/FTC + GSK3640254 (*n* = 15)	9,421.0 (1.15)	8,701.6–10,200.0	14.4	1,701 (1.23)	1,517.1–1,907.8	20.9	82.9 (1.33)	70.8–97.1	29.1	1.50 (1.00–3.08)

a AUC_0–τ_, area under the concentration-time curve from time zero to the end of the dosing interval at steady state; CI, confidence interval; *C*_max_, maximum observed concentration; *C*_τ_, concentration at the end of the dosing interval; %CVb, between-participant coefficient of variation; FTC, emtricitabine; ND, not described; PK, pharmacokinetic; SD ln, standard deviation of natural-logarithm-transformed data; TAF, tenofovir alafenamide; TFV, tenofovir; *T*_max_, time to first occurrence of *C*_max_.

**TABLE 3:** Pharmacokinetic parameter geometric least-squares mean ratios for TAF/FTC + GSK3640254 versus TAF/FTC^*a*^

Drug	GMR (90% CI) for:		
	AUC_0–τ_, ng·h/ml	*C*_max_, ng/ml	*C*_τ_, ng/ml
TAF	0.886 (0.75–1.04)	0.874 (0.68–1.12)	ND
TFV	1.036 (1.01–1.07)	1.018 (0.97–1.07)	1.073 (1.03–1.12)
FTC	0.963 (0.93–1.00)	0.941 (0.84–1.05)	1.163 (1.10–1.23)

a AUC_0–τ_, area under the concentration-time curve over 1 dosing interval; CI, confidence interval; *C*_max_, maximum observed concentration; *C*_τ_, concentration at the end of the dosing interval; FTC, emtricitabine; GMR, geometric least-squares mean ratio; ND, not described; TAF, tenofovir alafenamide; TFV, tenofovir.

Steady-state TFV and FTC PK were similar when TAF/FTC was administered alone or in combination with GSK3640254 (Tables 2 and 3). The between-participant variability across treatments was low for both TFV (range, 17.8% to 22.9%) and FTC (range, 14.4% to 29.1%). Plasma concentrations for TFV peaked with a median *T*_max_ of 3.0 h after dose administration and then declined slowly in a multiphasic manner (Fig. 1B). Median *T*_max_ for FTC occurred 1.5 h after administration, and then plasma FTC concentration declined in a biphasic manner (Fig. 1C). Steady state for both TFV and FTC was reached by day 11.

After TAF/FTC + GSK3640254 coadministration, GSK3640254 geometric mean steady-state exposure values (%CVb) for AUC_0–τ_, *C*_max_, and concentration at the end of the dosing interval (*C*_τ_) were 24.5 μg·h/ml (26.5), 1.4 μg/ml (26.4), and 0.8 μg/ml (30.3), respectively (Table S1). Maximum plasma concentrations of GSK3640254 peaked with a median *T*_max_ of 5.0 h after coadministration (Fig. 1D) and then declined in a monophasic manner. Steady state was reached by day 19.

### Safety and tolerability.

No deaths or serious AEs occurred. One participant withdrew because of a treatment-related grade 1 AE of urticaria during TAF/FTC + GSK3640254 coadministration on day 15; this AE resolved within 4 days after administration of diphenhydramine as a concomitant medication. A total of 9 participants (56%) reported 22 AEs during the study; 9 participants (56%) reported 19 AEs after TAF/FTC administration and 3 participants (19%) reported 3 AEs after TAF/FTC + GSK3640254 coadministration (Table 4). The most commonly reported AEs were upper respiratory tract infection (3 participants, 19%) and pustular rash and nausea (2 participants each, 13%), which were all reported after TAF/FTC administration. Other AEs were reported by 1 participant (6%) each. All AEs were grade 1 intensity, except for 1 participant who experienced 3 grade 2 AEs (1 occurrence of nausea and 2 occurrences of vomiting) after receiving TAF/FTC alone. There were no clinically relevant differences in laboratory values, electrocardiogram parameters, or vital signs between treatment groups.

**TABLE 4:** Summary of adverse events^*a*^

Preferred term	No. (%) in group with event	
	TAF/FTC (*n* = 16)	TAF/FTC + GSK3640254 (*n* = 16)
Any event	9 (56)	3 (19)
Upper respiratory tract infection	3 (19)	0
Rash, pustular	2 (13)	0
Nausea	2 (13)	0
Abdominal discomfort	1 (6)	0
Abnormal dreams	1 (6)	0
Back pain	1 (6)	0
Conjunctivitis	1 (6)	0
Headache	1 (6)	0
Ingrown hair	1 (6)	0
Lymphadenopathy	1 (6)	0
Muscular weakness	1 (6)	0
Rhinitis, allergic	1 (6)	1 (6)
Somnolence	0	1 (6)
Seasonal allergy	1 (6)	0
Urticaria	0	1 (6)
Vomiting	1 (6)	0

a FTC, emtricitabine; TAF, tenofovir alafenamide.

## DISCUSSION

The next-generation MI GSK3640254 could potentially be coadministered with the nucleoside reverse transcriptase inhibitors TAF/FTC. As *in vitro* studies suggested the potential for interactions between GSK3640254 and substrates of OATP1B3 such as TAF, this phase I trial evaluated the PK, safety, and tolerability of GSK3640254 and TAF/FTC, alone and in combination, in healthy participants. While clinically meaningful interactions were not expected, it is important to evaluate any potential interactions or changes in exposure with combination use.

After coadministration of GSK3640254 with TAF/FTC in the presence of food, TAF steady-state AUC_0–τ_ and *C*_max_ were slightly lower than when TAF/FTC was administered alone (11% and 13% lower, respectively), with GMRs (90% CIs) of 0.886 (0.75 to 1.04) and 0.874 (0.68 to 1.12), respectively. Although the lower boundaries of the 90% CIs (AUC_0–τ_, 0.75; *C*_max_, 0.68) were outside the 0.80-to-1.25 boundaries of no effect (https://www.fda.gov/media/134581/download), the decrease in TAF exposure observed in this study was comparable to that observed when 40 mg TAF daily (QD) (studied as TAF/FTC) is administered with 600 mg efavirenz QD (mean ratios [90% CI] for AUC and *C*_max_ were 0.86 [0.72 to 1.02] and 0.78 [0.58 to 1.05], respectively) (8). This magnitude of decrease is not considered clinically meaningful and does not require a dose adjustment (8).

Mean steady-state plasma exposure values for AUC_0–τ_, *C*_max_, and *C*_τ_ were similar when TAF/FTC was administered alone or in combination with GSK3640254. The 90% CIs for the ratios for these parameters were within the no-effect boundaries of 0.80 to 1.25. Because TFV is a metabolite of the TAF prodrug, the lack of effect on TFV exposure is supportive of no clinically meaningful interaction. Furthermore, median *T*_max_ values for plasma TAF, TFV, and FTC were unchanged when TAF/FTC and GSK3640254 were coadministered. After TAF/FTC + GSK3640254 coadministration, GSK3640254 steady-state plasma exposure values were comparable to those previously observed for GSK3640254 after 200-mg multiple-dose administration on day 14 (AUC_0–τ_, 21.5 μg·h/ml; *C*_max_, 1.40 μg/ml) with a similar median *T*_max_ of 3.8 h (range, 2 to 6) (7). These findings demonstrate that GSK3640254 is not associated with clinically meaningful effects on the steady-state PK of TAF, TFV, or FTC in healthy participants. Coadministration of GSK3640254 with TAF/FTC did not show any major safety or tolerability findings when given to healthy participants.

There are some limitations to this study. Sample sizes were small, the study was of short duration, and all participants were male, which limits generalizability. Furthermore, women of childbearing potential were not eligible because of the unknown effect of GSK3640254 on fetal development at the time the study was conducted.

GSK3640254 did not meaningfully affect the steady-state PK of TAF/FTC in healthy participants under fed conditions and did not show any major safety or tolerability findings when coadministered with TAF/FTC. These data support the upcoming phase IIb clinical trial in which GSK3640254 may be administered with TAF/FTC in treatment-naive adults with HIV-1 infection (NCT04493216).

## MATERIALS AND METHODS

### Study design.

This was a phase I, open-label, fixed-sequence, 2-period, 1-way drug interaction study to assess the PK, safety, and tolerability of TAF/FTC administered alone and in combination with GSK3640254 in healthy participants after a moderate-fat meal (ClinicalTrials.gov identifier, NCT03836729). The study consisted of a 28-day screening period and 2 sequential treatment periods with no washout between. Participants first received 25/200 mg TAF/FTC once daily (QD) on days 1 through 14 (period 1) and then received 25/200 mg TAF/FTC QD coadministered with 200 mg GSK3640254 QD on days 15 through 21 (period 2). The maximum projected clinical dose of 200 mg GSK3640254 QD and the currently approved dose of 25/200 mg TAF/FTC QD (8) were selected for this study. Participants fasted overnight for ≥8 h and received a moderate-fat meal 30 min before dosing, which occurred within 5 min of meal consumption. The study was conducted in accordance with the International Conference on Harmonization of Technical Requirements for Registration of Pharmaceuticals for Human Use Good Clinical Practice, following the principles of the Declaration of Helsinki. IntegReview Institutional Review Board (Austin, TX) approved the research protocol and study conduct.

### Study participants.

Eligible participants were healthy individuals aged 18 to 55 years with a body weight of ≥50 kg (men) or ≥45 kg (women) and a body mass index between 18.5 and 31.0 kg/m^2^. Women who were not of childbearing potential (i.e., those who were premenarchal, postmenopausal, or premenopausal if they had permanent infertility), pregnant, or breastfeeding were eligible for the study. Exclusion criteria were related to medical history (e.g., history of liver, gastrointestinal, cardiac, or psychiatric disorders) and laboratory screening tests, including a positive HIV, hepatitis B, or hepatitis C test. Participants were excluded if they had received a vaccine within 30 days or had prior or concomitant use of prescription or nonprescription treatments (including herbal or dietary supplements) that could affect the PK of the study drug. Other exclusion criteria included regular alcohol or tobacco use and sensitivity to study drugs.

### Assessments.

The primary endpoint was to assess the effect of GSK3640254 on the PK of TAF, tenofovir (TFV; a metabolite of TAF, which was measured to assess potential impacts on renal safety [8]), and FTC under fed conditions in healthy participants. Secondary endpoints included the assessment of safety and tolerability of TAF/FTC administered alone or in combination with GSK3640254 and characterization of the steady-state PK of TAF/FTC and TFV alone or in combination with GSK3640254. Blood samples for analysis of TAF, TFV, and FTC PK were collected before dosing on days 2 through 14 (period 1) and days 15 through 21 (period 2) and up to 24 h after TAF/FTC dosing on days 14 and 21. Samples for analysis of GSK3640254 were collected before dosing on days 16 through 21 and up to 24 h postdose on day 21. Safety and tolerability were assessed by monitoring and recording of adverse events (AEs), clinical laboratory test results (including platelet counts, white blood cell counts, hemoglobin and hematocrit measures, clinical chemistry assessments, and urinalysis), electrocardiogram results, and vital sign measurements (i.e., oral temperature, pulse rate, respiratory rate, and blood pressure).

### Bioanalytical methods.

Plasma concentrations of GSK3640254 were determined in human plasma via a validated ultra-high-performance liquid chromatography method with tandem mass spectrometry at PPD. GSK3640254 was extracted from 50 μl of human plasma by liquid-liquid extraction using methyl tert-butyl ether containing an isotopically labeled internal standard, [^2^H_4_^13^C_2_^15^N]GSK3640254. Chromatographic separation was achieved using a Waters CSH C_18_, 50- by 2.1-mm, 1.7-μm analytical column. The mobile phase consisted of 5 mM (pH 3) ammonium formate and acetonitrile/methanol, 90/10 (vol/vol). The flow rate was 0.7 ml/min, and the temperature was set at 65°C. The run time was ∼3.5 min. Transition mass was 727.6 → 179.1 for GSK3640254 and 734.6 → 186.2 for [^2^H_4_^13^C_2_^15^N]GSK3640254. The plasma assay range was 3.00 to 1,000 ng/ml.

Plasma concentrations of TAF were determined in human plasma via a validated high-performance liquid chromatography method with tandem mass spectrometry (HPLC-MS/MS) at PPD. Tenofovir alafenamide was extracted from 100 μl of human plasma by protein precipitation followed by phospholipid removal containing an isotopically labeled internal standard, TAF-d5. Chromatographic separation was achieved using a Waters Atlantis dC_18_, 2.1- by 50-mm, 3-μm analytical column. The mobile phase consisted of 1,000/1 (vol/vol) water-formic acid with 50/50 (vol/vol) acetonitrile/methanol. The flow rate was 0.4 ml/min at room temperature. The run time was ∼6 min. Transition mass was 477.1 → 346.2 for TAF and 482.2 → 351.3 for TAF-d5. The plasma assay range was 0.500 to 500 ng/ml.

Plasma concentrations of FTC were determined in human plasma via validated HPLC-MS/MS at PPD. Emtricitabine was extracted from 50 μl of human plasma by protein precipitation containing an isotopically labeled internal standard, [^13^C^15^N_2_]FTC. Chromatographic separation was achieved using a Waters Atlantis dC_18_, 2.1- by 50-mm, 3-μm analytical column. The mobile phase consisted of 1,000/1 (vol/vol) water-formic acid with 50/50 (vol/vol) acetonitrile/methanol. The flow rate was 0.300 ml/min at room temperature. The run time was ∼6 min. The transition mass was 248.1 → 130.1 for FTC and 251.1 → 133.1 for [^13^C^15^N_2_]FTC. The plasma assay range was 20.0 to 4,000 ng/ml.

Quality control (QC) samples containing GSK3640254 (3 concentrations), TAF (5 concentrations), or FTC (5 concentrations) and stored with study samples were analyzed with each batch of samples against separately prepared calibration standards. For analyses to be acceptable, no more than one-third of the total QC results and no more than one-half of the results from each concentration level were to deviate from the nominal concentration by >15%. Applicable analytical runs met all predefined run acceptance criteria, and assays performed robustly during plasma sample analysis. All calibration lines had correlation coefficient (*r*^2^) values of >0.998, >0.997, and >0.999 for GSK3640254, TAF, and FTC, respectively. Coefficient-of-variation values for QC samples ranged from 3.11% to 4.41% for GSK3640254, 5.73% to 8.70% for TAF, and 4.64% to 11.7% for FTC. Run-to-run mean bias values for QC samples ranged from 1.42% to 2.25% for GSK3640254, 1.39% to 4.54% for TAF, and −2.56% to 1.15% for FTC.

### Data analyses.

A sample size of 12 participants was deemed sufficient to account for the intra- and interparticipant variability to evaluate safety and tolerability and to estimate PK parameters. Approximately 16 participants were enrolled to ensure that 12 evaluable participants completed the study. Pharmacokinetic parameters were calculated by standard noncompartmental analysis using WinNonlin (version 8.0). The primary plasma PK endpoints included area under the plasma concentration-time curve from time zero to the end of the dosing interval at steady state (AUC_0–τ_), maximum observed concentration (*C*_max_), and plasma concentration at the end of the dosing interval (*C*_τ_). The plasma PK parameters AUC_0–τ_ and *C*_max_ were estimated for TAF, and AUC_0–τ_, *C*_max_, and *C*_τ_ were also estimated for TFV and FTC. Plasma PK parameter values, including arithmetic mean, geometric mean, median, standard deviation, minimum, maximum, and coefficient of variation, were summarized by treatment. Secondary plasma PK parameters (*T*_max_) were summarized using descriptive statistics. Analyses were performed on natural logarithms of AUC_0–τ_, *C*_max_, and *C*_τ_ using linear mixed-effect models with period as a fixed effect, participant as a random effect, and measurements within participant as repeated measures. Geometric least-squares mean ratios (GMRs) and 90% CIs were derived for comparisons of period 2 versus period 1. Safety endpoints were summarized using descriptive statistics.

Anonymized individual participant data and study documents can be requested for further research from www.clinicalstudydatarequest.com.
