# Supplementary material for: A Phase I Evaluation of the Pharmacokinetics and Tolerability of the HIV-1 Maturation Inhibitor GSK3640254 and Tenofovir Alafenamide/Emtricitabine in Healthy Participants
Source: Antimicrob Agents Chemother. 2021 May 18;65(6):e02173-20. doi: 10.1128/AAC.02173-20 (PMC8316023; doi:10.1128/AAC.02173-20)
Supplement: Supplementary file 1 [file aac.02173-20-s0001.pdf]

TABLE S1. Summary Statistics of Derived GSK3640254 Plasma PK Parameters

PK parameter	TAF/FTC + GSK3640254 (N=15)
$AUC_{0-\tau}$, geometric mean (SD ln), h*μg/mL	24.5 (1.30)
95% CI	21.2-28.3
%CVb	26.5
C_{max}, geometric mean (SD ln), μg/mL	1.4 (1.30)
95% CI	1.2-1.6
%CVb	26.4
C_{τ}, geometric mean (SD ln), μg/mL	0.8 (1.34)
95% CI	0.7-0.9
%CVb	30.3
T_{max}, median (range), h	5.0 (2.50-8.00)

$AUC_{0-\tau}$, area under the concentration-time curve from time 0 to the end of the dosing interval at steady state; C_{max}, maximum observed concentration; C_{τ}, concentration at the end of the dosing interval; %CVb, between-participant coefficient of variation; FTC, emtricitabine; ln, natural logarithm; PK, pharmacokinetic; SD ln, standard deviation of ln-transformed data; TAF, tenofovir alafenamide; TFV, tenofovir; T_{max}, time to first occurrence of C_{max}.

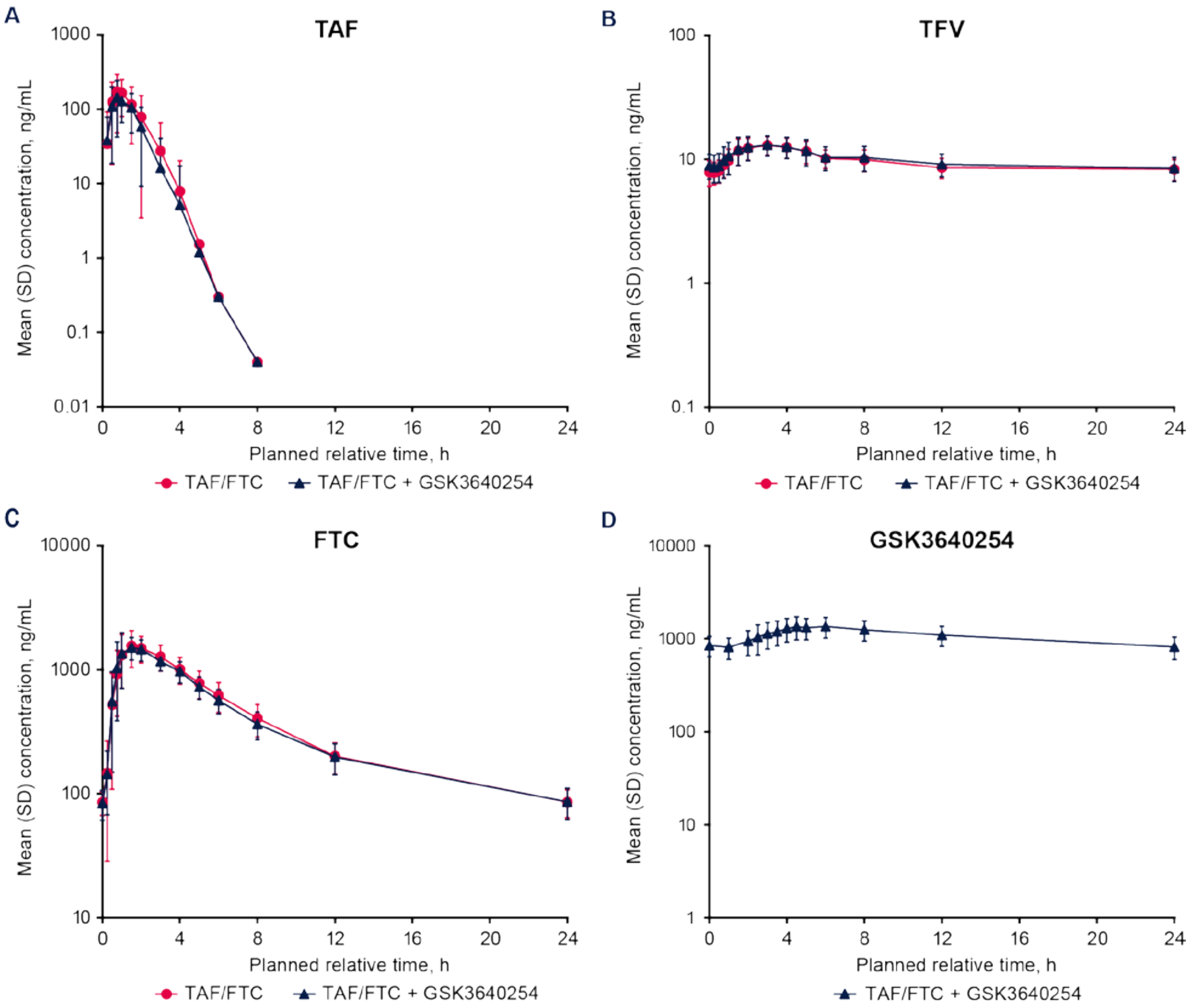

FIG S1. Mean semi-logarithmic plasma concentration-time profiles at steady state for **(A)** TAF, **(B)** TFV, **(C)** FTC, and **(D)** GSK3640254. Error bars represent SD. Non-quantifiable concentrations were imputed; if >30% of values were imputed, then the SD was not represented. FTC, emtricitabine; SD, standard deviation; TAF, tenofovir alafenamide; TFV, tenofovir.

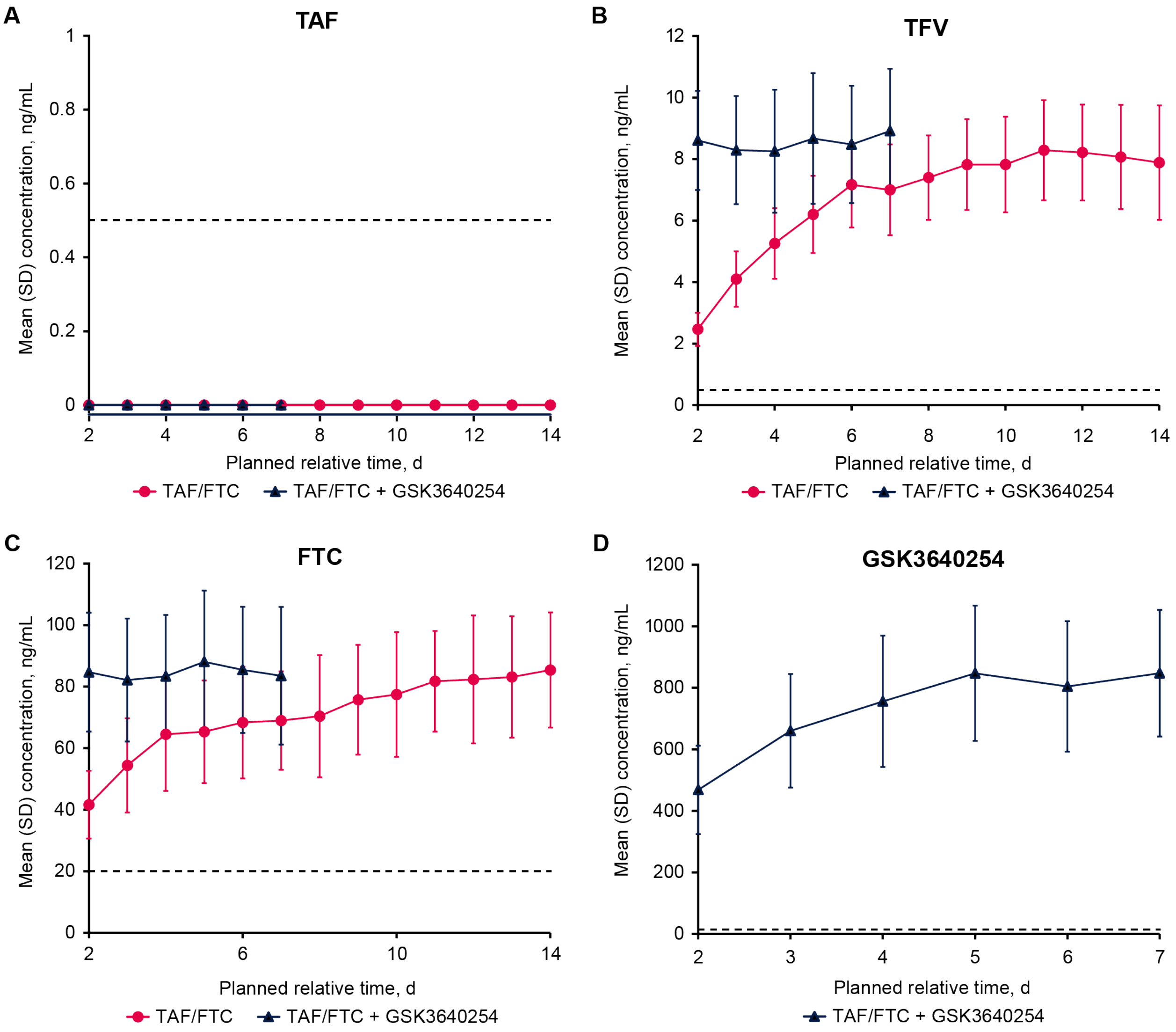

FIG S2. Summary of predose (trough) mean plasma concentration-time profiles for **(A)** TAF, **(B)** TFV, **(C)** FTC, and **(D)** GSK3640254. Error bars represent SD. Non-quantifiable concentrations were imputed with 0. Dashed lines represent LLOQ. LLOQ = 0.5 ng/mL for TAF and TFV, 20.0 ng/mL for FTC, and 3.0 ng/mL for GSK3640254. All TAF predose concentrations were below LLOQ. d, day relative to the start of Period 1 (for TAF/FTC) or Period 2 (for TAF/FTC + GSK3640254); FTC, emtricitabine; LLOQ,

lower limit of quantification; SD, standard deviation; TAF, tenofovir alafenamide; TFV, tenofovir.
